# A potential prognostic prediction model of colon adenocarcinoma with recurrence based on prognostic lncRNA signatures

**DOI:** 10.1186/s40246-020-00270-8

**Published:** 2020-06-10

**Authors:** Lipeng Jin, Chenyao Li, Tao Liu, Lei Wang

**Affiliations:** Department of Colorectal & Anal Surgery, First Hospital Bethune of Jilin University, No. 71, Xinmin Street, Chaoyang District, Changchun, Jilin, 130000 China

**Keywords:** Colon adenocarcinoma, Recurrence, Differentially expressed genes, Prognosis

## Abstract

**Background:**

Colon adenocarcinoma (COAD) is one of the common gastrointestinal malignant diseases, with high mortality rate and poor prognosis due to delayed diagnosis. This study aimed to construct a prognostic prediction model for patients with colon adenocarcinoma (COAD) recurrence.

**Methods:**

Differently expressed RNAs (DERs) between recurrence and non-recurrence COAD samples were identified based on expression profile data from the NCBI Gene Expression Omnibus (GEO) repository and The Cancer Genome Atlas (TCGA) database. Then, recurrent COAD discriminating classifier was established using SMV-RFE algorithm, and receiver operating characteristic curve was used to assess the predictive power of classifier. Furthermore, the prognostic prediction model was constructed based on univariate and multivariate Cox regression analysis, and Kaplan-Meier survival curve analysis was used to estimate this model. Furthermore, the co-expression network of DElncRNAs and DEmRNAs was constructed followed by GO and KEGG pathway enrichment analysis.

**Results:**

A total of 54 optimized signature DElncRNAs were screened and SMV classifier was constructed, which presented a high accuracy to distinguish recurrence and non-recurrence COAD samples. Furthermore, six independent prognostic lncRNAs signatures (LINC00852, ZNF667-AS1, FOXP1-IT1, LINC01560, TAF1A-AS1, and LINC00174) in COAD patients with recurrence were screened, and the prognostic prediction model for recurrent COAD was constructed, which possessed a relative satisfying predicted ability both in the training dataset and validation dataset. Furthermore, the DEmRNAs in the co-expression network were mainly enriched in glycan biosynthesis, cardiac muscle contraction, and colorectal cancer.

**Conclusions:**

Our study revealed that six lncRNA signatures acted as an independent prognostic biomarker for patients with COAD recurrence.

## Introduction

As one of the most common gastrointestinal malignant diseases, colon adenocarcinoma (COAD) is the world-wide leading cause of mortality [1]. Currently, the standard therapeutic method for COAD is the combination of surgery and adjuvant chemotherapy or radiation therapy [2]. Additionally, the early diagnosis for primary or recurrent COAD is also a critical factor for improving the prognosis of patients [3]. Unfortunately, despite substantial advances in early diagnosis and treatments, poor survival, high recurrence, and unsatisfactory prognosis remain an issue due to delayed diagnosis and adverse drug effects [2, 4]. Therefore, identification of novel diagnostic, prognostic biomarkers and therapeutic targets, as well as investigation of the underlying molecular mechanism of COAD, is required.

Ideal diagnostic and prognostic biomarkers should be strongly associated with the prognosis of patients and easy to detect [5]. Encouragingly, overwhelming evidence has demonstrated that the regulatory roles of noncoding RNAs such as long non-coding RNAs (lncRNAs) are predominately correlated with the development and progression of a wide variety of cancers [6]. LncRNAs are arbitrarily defined as noncoding RNA with the length > 200 nucleotides, and have several possible functions, including miRNA sponges, regulating gene transcription and splicing, and forming RNA-protein complexes [7]. It is now well appreciated that lncRNAs participate in disease progression by regulating various key cell biological processes such as cell proliferation, differentiation, apoptosis, migration, and invasion [8]. To date, lncRNAs have been revealed abnormal expression in cancers, and some of which are served as oncogenes or tumor suppressors [8]. Furthermore, accumulating studies have shown that lncRNAs are potentially identified as novel diagnostic, prognostic, and metastasis predictive biomarkers in various cancers [9–11]. Recently, several lncRNA profiling has identified several colorectal cancer-specific lncRNAs, and the following experiments have demonstrated that lncRNAs such as PCAT-1, RP11-462C24.1, HOTAIR, and MALAT1 are candidate diagnostic biomarkers [12–14]. However, few studies have investigated lncRNAs as the prognostic biomarkers for recurrent COAD.

In the current study, lncRNAs related to COAD recurrence were screened based on expression profile data from the National Center for Biotechnology Information (NCBI) GEO repository and The Cancer Genome Atlas (TCGA). Next, a recurrent COAD discriminating classifier and a prognostic prediction model were constructed using the bioinformatics methods. Moreover, co-expression network and pathways were analyzed. According to this, we aimed to explore a useful prognostic prediction model for recurrent COAD and provide some useful insights in improving the prognosis of recurrent COAD patients.

## Materials and methods

### Data extraction and preprocessing

The gene expression datasets were preliminarily extracted from the NCBI GEO repository (https://www.ncbi.nlm.nih.gov/geo/) using search words of “colon adenocarcinoma and *Homo sapiens*”. Then, the eligible dataset were selected in this study according to the following criteria: (1) the samples in datasets were solid tissues of COAD patients; (2) the total number of COAD samples was not less than 500; and (3) the datasets contained recurrence and prognosis information of samples. Eventually, GSE39582 was obtained and utilized as training dataset. This dataset was generated from GPL570 Affymetrix Human Genome U133 Plus 2.0 Array platform and contained 585 COAD samples and 574 samples had recurrence information [15].

Meanwhile, the RNA sequencing data and corresponding clinical information of COAD patients were downloaded from TCGA (https://gdc-portal.nci.nih.gov/). This dataset was obtained from the platform of Illumina HiSeq 2000 RNA Sequencing and contained 512 COAD samples. After the RNA sequencing data was matched with clinical information, a total of 310 samples containing recurrence and prognosis information were obtained, which was utilized as validation dataset. The clinical characteristics of COAD patients in the training and validation datasets are shown in Table 1.

**Table 1:** Clinical characteristics of the colon adenocarcinoma samples in the training and validation datasets

Clinical characteristics	Training dataset (N=574)	Validation dataset (N=310)
Age at pathologic diagnosis (years, mean ± sd)	66.89 ± 13.22	65.73 ± 12.71
Gender (male/female)	317/257	169/141
Pathologic stage (1/2/3/4)	41/267/206/60	51/118/88/43/10
Pathological T (1/2/3/4/–)	13/47/373/118/23	8/55/212/34/1
Pathological N (0/1/2/3/–)	309/134/99/6/26	180/77/53/0/0
Pathological M (0/1/-)	491/61/22	226/43/41
Tumor location (distal/proximal)	348/226	–
Chemotherapy (yes/no)	239/319/16	–
Tumor recurrence (yes/no)	179/375	66/244
Recurrence free survival time (months, mean ± sd)	49.79 ± 40.76	29.66 ± 25.46

### Screening of differentially expressed RNAs (DERs)

Firstly, all mRNAs and lncRNAs in training and validation dataset were annotated based on the HUGO Gene Nomenclature Committee (HGNC, http://www.genenames.org/) database [16], consisting of annotated 19,198 protein coding genes and 4120 lncRNAs. Then, the overlapping mRNAs and lncRNAs were obtained between these two datasets. All COAD samples in the training dataset were divided into recurrence and non-recurrence groups. The limma package (version 3.34.7, https://bioconductor.org/packages/release/bioc/html/limma.html) [17] in R 3.4.1 was utilized to screen DERs (including mRNA and lncRNA) between recurrence and non-recurrence samples with the thresholds of false discovery rate < 0.05 and |log_2_ fold change| > 0.263. Furthermore, bidirectional hierarchical clustering based on centered Pearson correlation algorithm [18] was performed by pheatmap (Version 1.0.8, https://cran.r-project.org/web/packages/pheatmap/index.html) [19] in R 3.4.1 according to the expression values of DERs in the training dataset.

### Screening of signature lncRNAs

The e1071 (Version 1.7-1, https://cran.r-project.org/web/packages/e1071) [20] and caret package (Version 6.0-76, https://cran.r-project.org/web/packages/caret) [21] in R was used to identify optimized signature lncRNAs based on recursive feature elimination (RFE) algorithm. Next, the SVM-based classifier was built to predict COAD recurrence based on signature lncRNAs. In addition, the performance of the classifier was evaluated in the training dataset and validation dataset, respectively. The area under curve (AUC) index was calculated to evaluate the predictive power of the classifier based on receiver operating characteristic (ROC) curve analysis using pROC (Version 1.15.0, https://cran.r-project.org/web/packages/pROC/index.html) [22] in R. The corresponding parameters, including sensitivity (Sen), specificity (Spe), positive prediction value (PPV), and negative prediction value (NPV), were also calculated using pROC.

### Constructions and verification of prognostic prediction model

The univariate cox regression analysis for the lncRNAs used for SVM classifier construction was carried out using survival package (Version 2.41.1, http://bioconductor.org/packages/survivalr/) [23] in R3.4.1 with the threshold of log-rank *P* value < 0.05. Then, independent prognostic lncRNAs were further screened by multivariate cox regression analysis using survival package (Version 2.41.1) [23]. Afterwards, the risk score (RS) prognostic prediction model was constructed based on expression levels of independent prognostic lncRNAs and their regression coefficients estimated from the multivariate Cox regression model as follows: RS = ∑β_lncRNA_ × Exp_lncRNA_. The β_lncRNA_ represented the independent prognostic coefficient and Exp_lncRNA_ was defined as the expression value of corresponding lncRNA. According to the median value of RS, all samples in the training dataset were divided into high-risk and low-risk groups. The Kaplan-Meier (K-M) survival curve analysis was performed to evaluate survival difference between high- and low-risk group using survival package (version 2.41.1) in R 3.4.1. Moreover, the prognostic significance of RS was also assessed by including C-index [24], Brier score [25], and log-rank *P* value of cox-PH regression [26]. Similarly, a RS model was also established in validation set. Accordingly, K-M curves were constructed to analyze two risk groups and COAD survival. C-index, Brier score, and log-rank *P* value C-index were used to evaluate the predictive accuracy of RS.

### Construction of co-expression network and functional analysis

The expression levels of the signature lncRNAs and differentially expressed mRNAs were extracted from COAD samples in the training dataset. Next, co-expression network was constructed based on Pearson correlation coefficient (PCC) of the prognostic lncRNAs and DE mRNAs using cor.test function (https://stat.ethz.ch/R-manual/R-devel/library/stats/html/cor.test.html) [27] in R3.4.1 and then visualized by Cytoscape (Version 3.6.1, https://cytoscape.org/). Besides, Gene Ontology (GO) functional annotation associated with biological process analysis as well as Kyoto Encyclopedia of Genes and Genomes (KEGG) pathway enrichment analysis of mRNAs in co-expression network were performed based on Database for Annotation, Visualization, and Integrated Discovery (DAVID) program (v 6.8, https://david.ncifcrf.gov/) [28, 29] with the threshold of *P* value < 0.05.

## Results

### DERs screening between recurrence and non-recurrence COAD samples

**Fig. 1:**
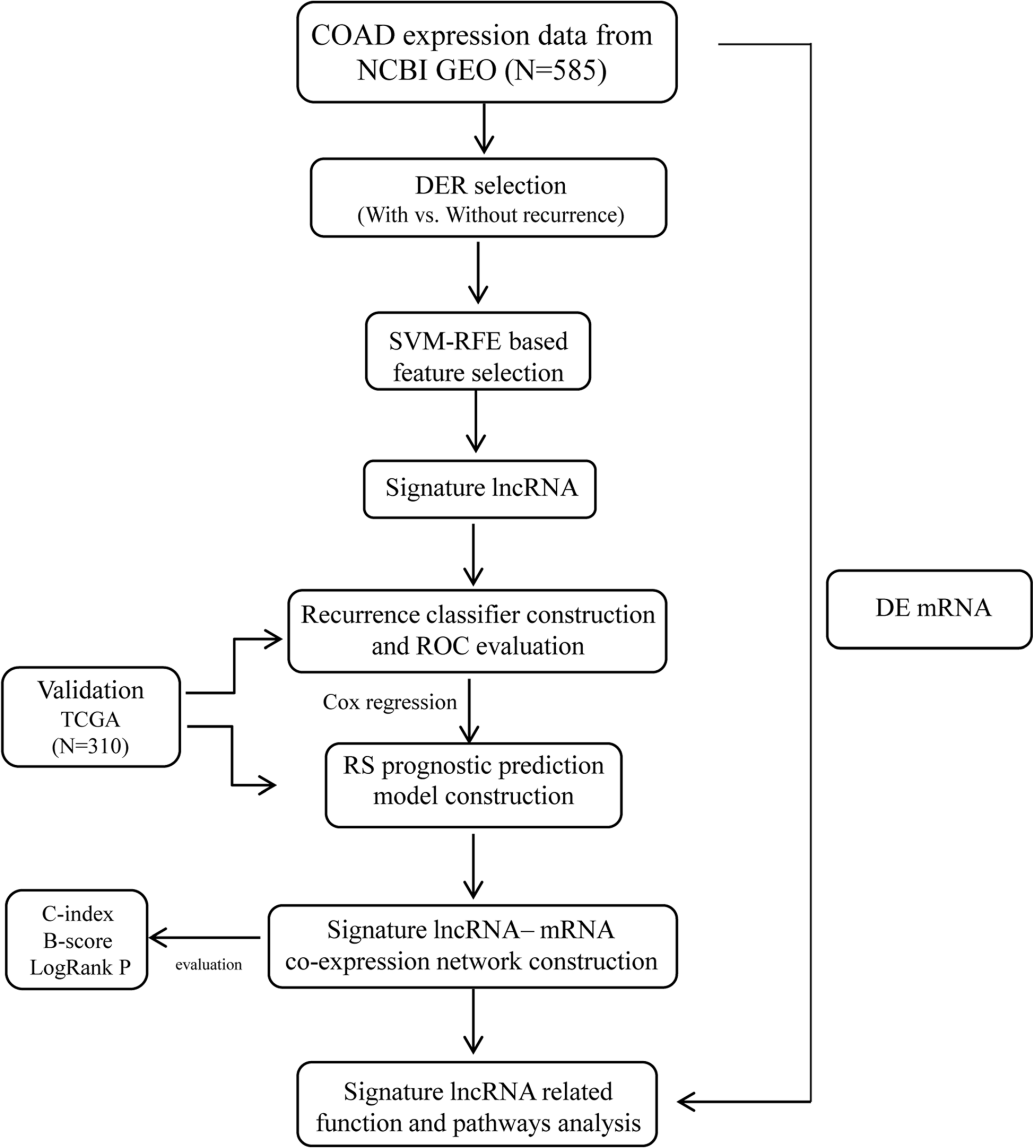
Workflow of this study

**Fig. 2:**
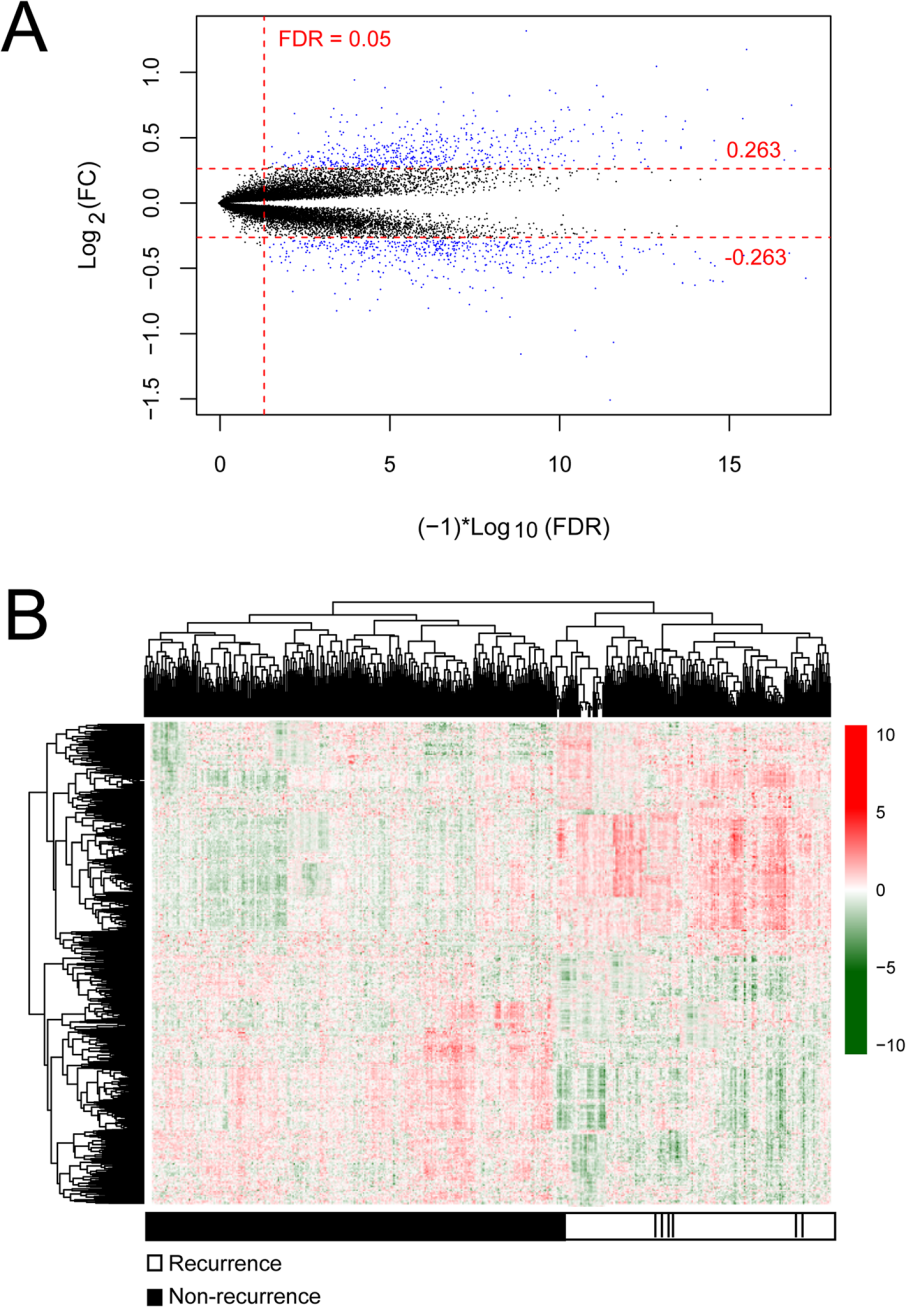
Identification of differentially expressed RNAs (DERs). **a** Volcano map. Blue dots indicate DERs, the red horizontal dotted line represents false discovery rate (FDR) < 0.05, the two red vertical dotted lines represent the |log fold change (FC)| > 0.263. **b** A bidirectional hierarchical clustering map based on DERs. White and black sample bars represent recurrence and non-recurrence colon adenocarcinoma samples, respectively

This study was conducted as indicated in Fig. 1. After annotation, there were 13834 mRNAs and 827 lncRNAs between training and validation dataset. Subsequently, a total of 1002 DERs were identified between recurrence (*n* = 179) and non-recurrence (*n* = 375) COAD samples based on the selective criteria, including 939 DE mRNAs (475 downregulated and 464 upregulated mRNAs) and 63 DE lncRNAs (13 downregulated and 50 upregulated lncRNAs) (Fig. 2a). The bidirectional hierarchical clustering analysis indicated that these DERs could significantly distinguish recurrent and non-recurrent COAD samples (Fig. 2b).

### Signature lncRNA screening

**Fig. 3:**
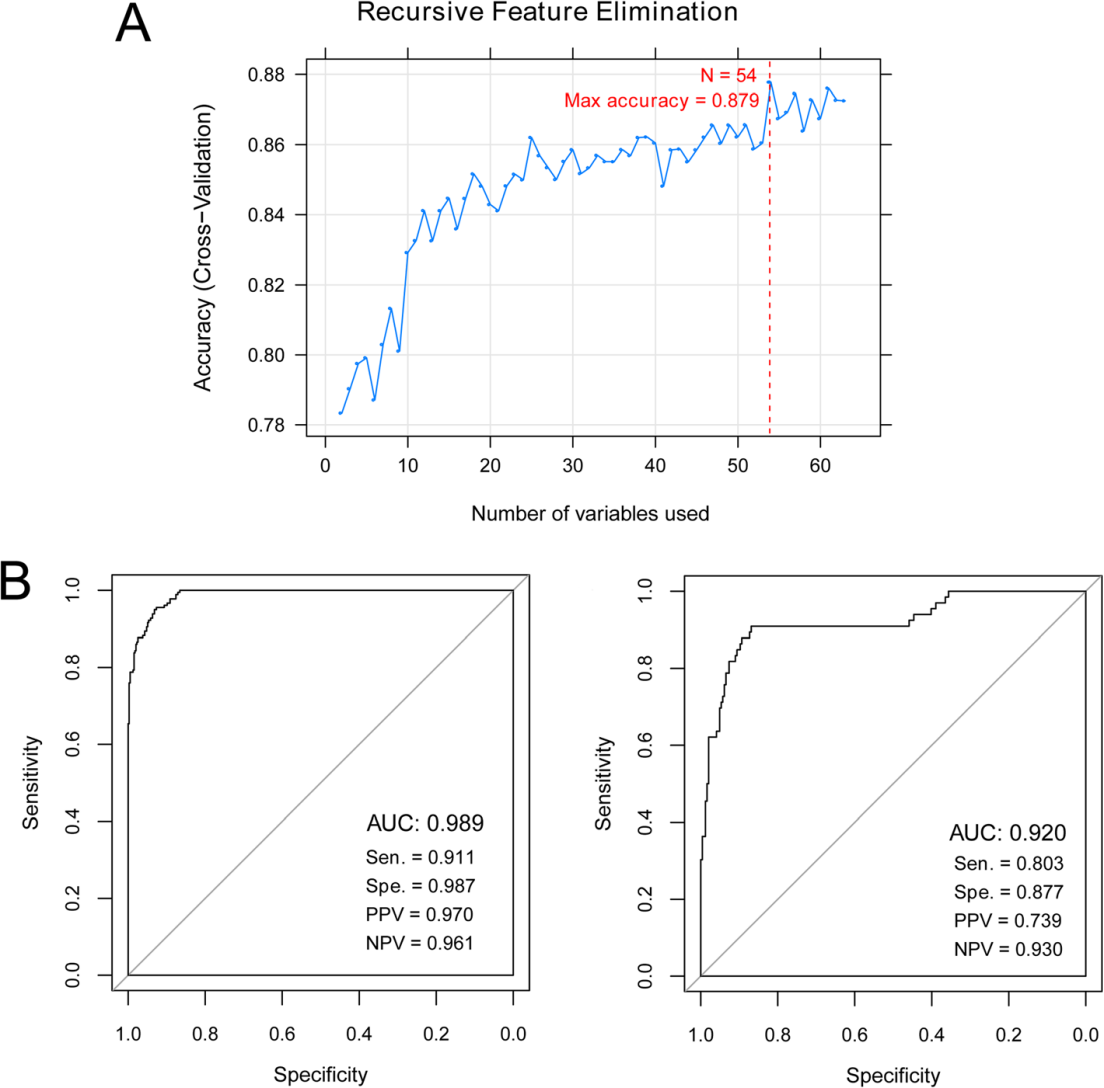
Screening of signature lncRNAs and construction of recurrence colon adenocarcinoma (COAD) discriminating classifier. **a** Accuracy curve of the optimized signature lncRNAs using recursive feature elimination (RFE) algorithm. **b** Receiver operating characteristic (ROC) curve based on the support vector machine (SVM) classifier in the training dataset (left) and validation dataset (right)

The SVM-RFE algorithm was used to identify the most optimized lncRNA signatures. We found that there were 54 optimized lncRNA signatures when the accuracy was the highest value of 0.879 (Fig. 3a). Then, a SMV classifier was established based on the 54 optimized lncRNAs to differentiate recurrent COAD samples from non-recurrent COAD samples. ROC curve analysis revealed that this SVM classifier exhibited a good discriminatory power for patients with or without COAD recurrence in training dataset (AUC 0.989, Sen 0.911, Spe 0.987, PPV 0.970, and NPV 0.961; Fig. 3b). Similarly, a SVM-based classifier was also built in validation set and it had a high accuracy to distinguish recurrent and non-recurrent COAD samples (AUC 0.920, Sen 0.803, Spe 0.877, PPV 0.739, and NPV 0.930; Fig. 3b).

### Construction and verification of prognostic prediction model

**Fig. 4:**
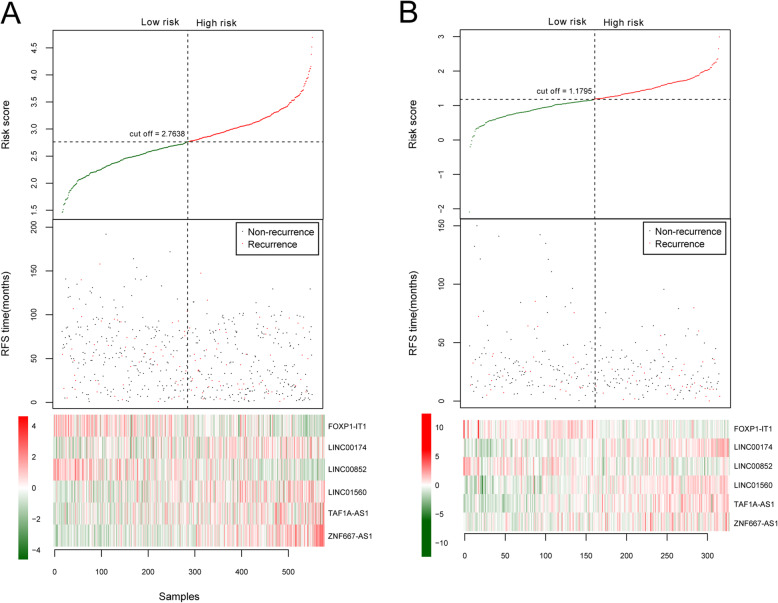
A lncRNA-based risk score model in the training and validation dataset. **a** The training dataset. **b** The validation dataset. The top row in each pane shows the distribution of risk score distribution. The middle row in each pane shows the survival status of colon adenocarcinoma patients. The bottom row in each pane shows the heatmap of the expression of the 6 key lncRNAs. The color, from green to red, shows low to high expression

**Fig. 5:**
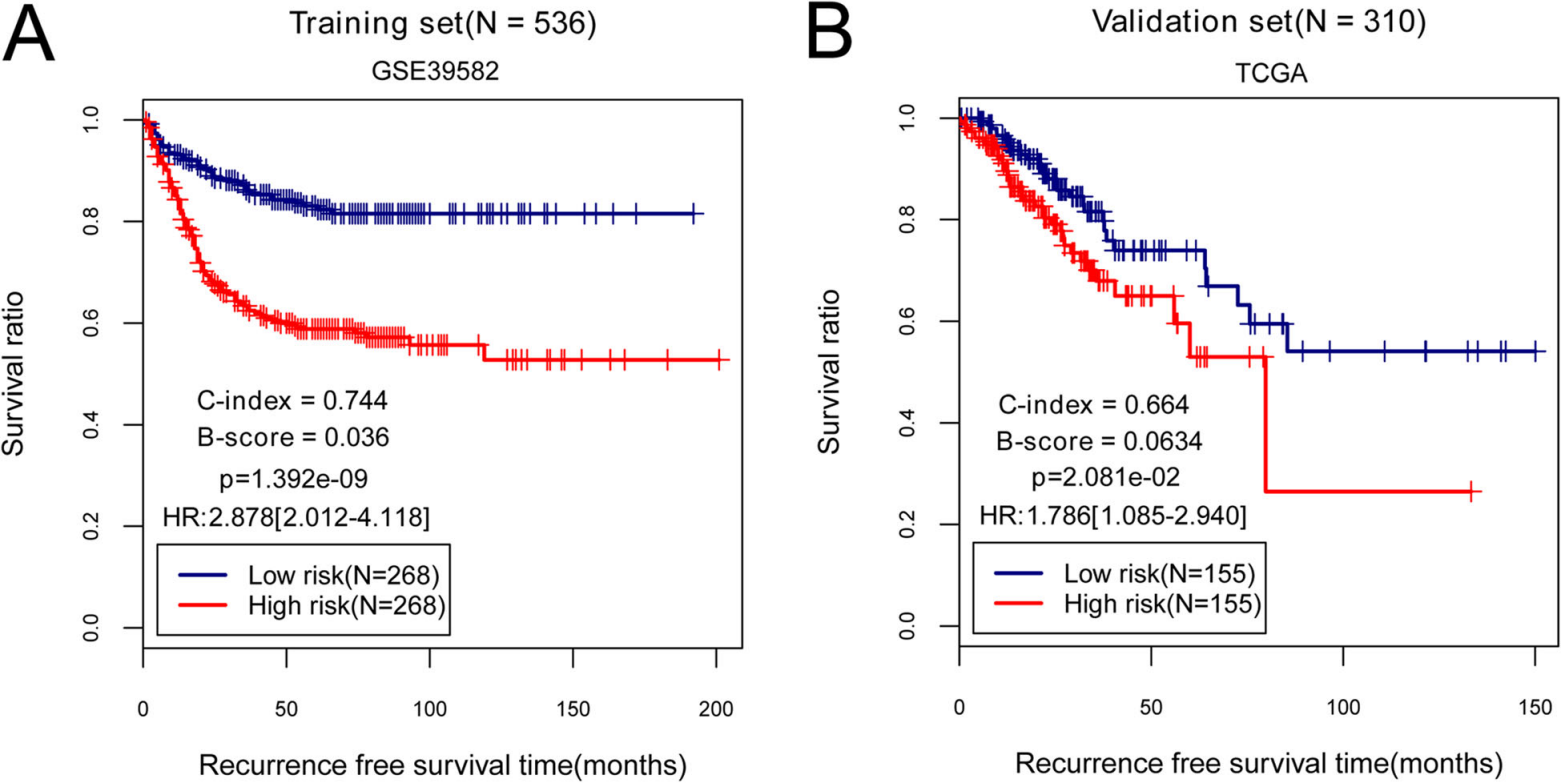
Performance evaluation of the risk score (RS) prognostic prediction model. **a** K-M survival analysis based on RS prognostic prediction model in the training dataset. **b** K-M survival analysis based on RS prognostic prediction model in the validation dataset

Based on univariate Cox regression analysis in the training dataset, a total of 39 DElncRNAs were significantly associated with the overall survival of patients with COAD recurrence. Then, six independent prognostic lncRNAs (LINC00852, ZNF667-AS1, FOXP1-IT1, LINC01560, TAF1A-AS1, and LINC00174) were obtained by multivariate Cox regression analysis (Table 2). Among them, two lncRNAs (LINC00852, FOXP1-IT1) with negative coefficients revealed that their higher expressions were correlated with longer survival, while the remaining lncRNAs (ZNF667-AS1, LINC01560, TAF1A-AS1, and LINC00174) with positive coefficients indicated that their higher expression were associated with unfavorable survival outcomes. Next, the RS prognostic prediction model was constructed based on the coefficients of six independent prognostic lncRNAs and their expression levels in training dataset as follows: RS = (− 0.9109) × Exp_LINC00852_ + (0.4241) × Exp_ZNF667-AS1_ + (− 0.4840) × Exp_FOXP1-IT1_ + (0.3945) × Exp_LINC01560_ + (0.4466) × Exp_TAF1A-AS1_ + (0.6742) × Exp_LINC00174_. Subsequently, the RS of each patient was calculated and all patients were then divided into high-risk group (*n* = 268) and low-risk group (*n* = 268) according to the median value of RS (Table 3). Accordingly, the distribution of the RS and survival status of the GC patients as well as the expression levels of six prognostic lncRNA signatures were also obtained. As displayed in Fig. 4a, there were high expressions of risky lncRNAs (ZNF667-AS1, LINC01560, TAF1A-AS1, and LINC00174) in patients from the high-risk group. Conversely, those patients in the low-risk group tended to express high levels of protective lncRNAs (LINC00852, FOXP1-IT1). These findings were verified in the validation dataset as shown in Fig. 4b. Additionally, the effects of six prognostic lncRNAs on survival outcomes of COAD patients in high- and low-risk group were also assessed by K-M analysis in training and validation sets. The results suggested that there was a lower survival probability for COAD patients in the high-risk group than that in the low-risk group (training dataset: a log-rank *P* value = 1.392e−09, HR = 2.878, 95%CI 2.012–4.118, C-index = 0.744, and Brier score = 0.036; validation dataset: a log-rank *P* value = 2.081e−02, HR = 1.786, 95%CI 1.085–2.940, C-index = 0.664, and Brier score = 0.063; Fig. 5).

**Table 2:** Six independent prognostic lncRNAs in colon adenocarcinoma patients with recurrence

ID	Coef	P value	Hazard ratio	95%CI
LINC00852	− 0.9109	2.73E−03	0.4022	0.2217–0.7296
ZNF667-AS1	0.4241	1.68E−02	1.5282	1.0794–2.1638
FOXP1-IT1	− 0.4840	2.32E−02	0.6163	0.4059–0.9359
LINC01560	0.3945	2.85E−02	1.4836	1.0424–2.1115
TAF1A-AS1	0.4466	3.90E−02	1.5630	1.0229–2.3884
LINC00174	0.6742	4.31E−02	1.9624	1.0210–3.7718

**Table 3:** Independent prognostic factors of colon adenocarcinoma patient with recurrence by univariate and multivariate Cox regression analysis

Clinical characteristics	Univariable Cox			Multivariable Cox		
	HR	95%CI	P	HR	95%CI	P
Age at pathologic diagnosis (years, mean ± sd)	1.011	0.997–1.023	1.268E−01	–	–	–
Gender (male/female)	1.309	0.940–1.823	1.096E−01	–	–	–
Pathologic stage (1/2/3/4)	1.866	1.475–2.360	1.754E−07	1.365	0.899–2.073	1.439E−01
Pathological T (1/2/3/4/–)	1.969	1.443–2.686	2.606E−05	1.686	1.199–2.369	2.630E−03
Pathological N (0/1/2/3/–)	1.691	1.387–2.059	5.525E−07	1.328	0.967–1.824	7.921E−02
Pathological M (0/1/–)	1.77	0.861–3.639	1.154E−01	–	–	–
Tumor location (distal/proximal)	0.346	0.602–1.181	3.217E−01	–	–	–
Chemotherapy (yes/no)	1.647	1.190–2.277	2.331E−03	0.998	0.669–1.490	9.922E−01
RS model status (high/low)	2.878	2.012–4.118	1.392E−09	2.559	1.747–3.748	1.410E−06
Tumor recurrence (yes/no)	–	–	–	–	–	–
Recurrence free survival time (months, mean ± sd)	–	–	–	–	–	–

### Construction of co-expression network and functional analysis

**Fig. 6:**
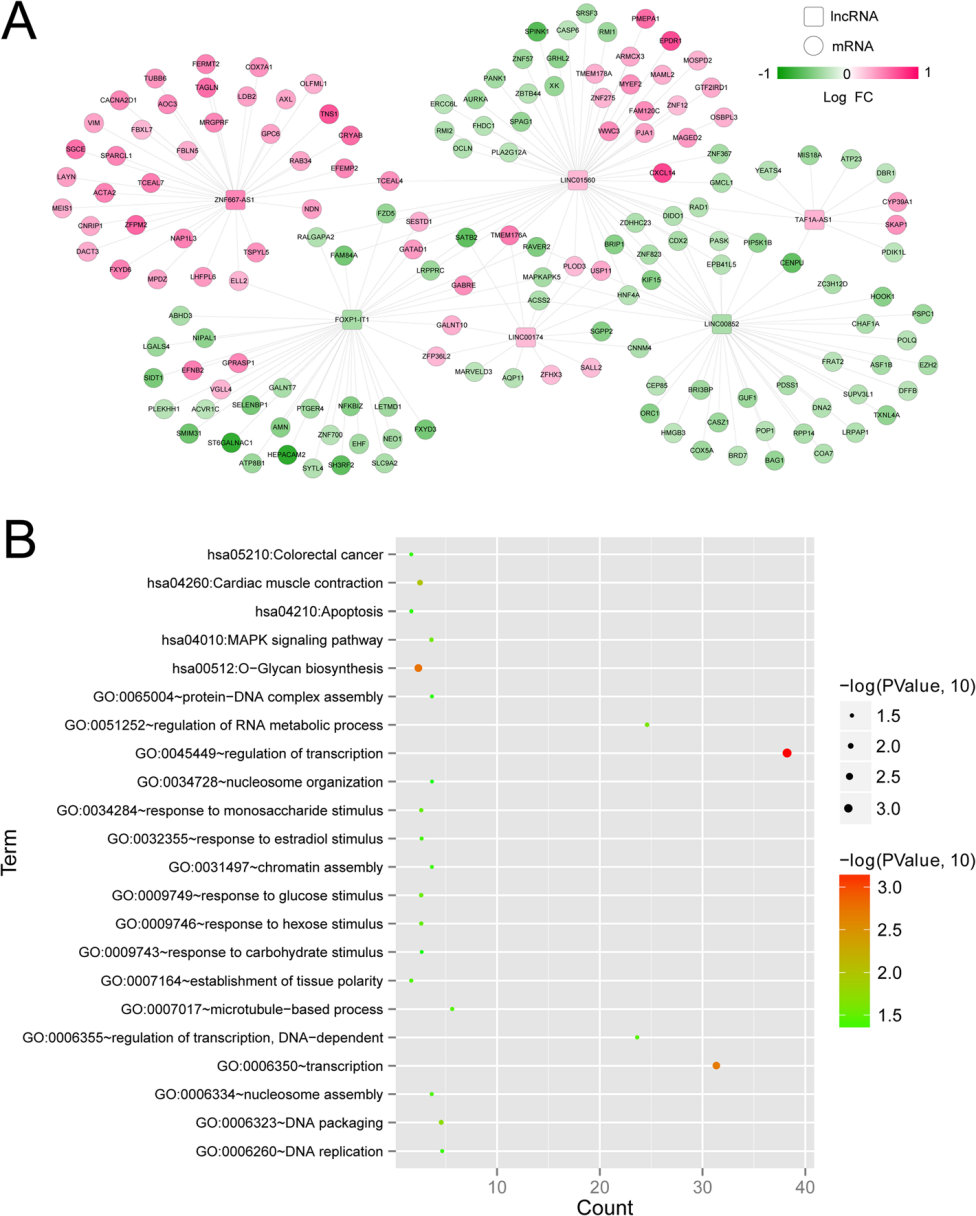
Construction of co-expression network and functional analysis. **a** The co-expression network of differentially expressed (DE) lncRNAs and DEmRNAs. The red color shows the upregulated lncRNAs or mRNAs, and the green color denotes the downregulated lncRNAs or mRNAs. Squares and circles, respectively, represent lncRNAs and mRNAs. **b** GO and KEGG pathways of DEmRNAs. Horizontal axis and vertical axis represent the gene number and term, respectively; the color and size of the bots indicate the significant *P* value, and the closer the color is to red, the higher the significance

The co-expression analysis between prognostic lncRNAs and DEmRNAs was performed. There were 198 DE lncRNA-DE mRNA pairs among 6 lncRNAs, and 162 DE mRNAs (such as *CXCL14*, *EPDR1*, *PMEPA1*, *HEPACAM*, *ST6GALNAC1*, and *SELENBP1*) were obtained (Fig. 6a). Subsequently, the functional analyses of DEmRNAs in co-expression network were conducted and the results revealed that these genes were significantly enriched in 17 GO-BP terms such as regulation of transcription, and 5 KEGG pathways, including glycan biosynthesis, cardiac muscle contraction, MAPK signaling pathway, colorectal cancer, and apoptosis (Fig. 6b).

## Discussion

In this study, 939 DEmRNAs and 63DElncRNAs were identified between recurrent and non-recurrent COAD. Then, 54 optimized signature DElncRNAs were screened and subjected to a SMV classifier construction. Furthermore, 6 independent prognostic lncRNAs signatures (LINC00852, ZNF667-AS1, FOXP1-IT1, LINC01560, TAF1A-AS1, and LINC00174) were screened by univariate and multivariate Cox regression analysis, and the RS prognostic prediction model for recurrent COAD was constructed and showed a high predictive value for COAD samples both in the training dataset and validation dataset. Furthermore, the co-expression network revealed that LINC01560 interacted with *CXCL14* and *EPDR1* while FOXP1-IT1 was co-expressed with *HEPACAM* and *ST6GALNAC1*.

The mining of accumulating gene expression profile data in a variety of diseases have been enhanced with the rapid advances in high-throughput sequencing and bioinformatics technologies [30]. This study integrated the eligible expression profile data of COAD samples with and without recurrence from NCBI GEO and screened 63 DElncRNAs related to COAD recurrence. As a powerful classification tool, SVM is widely applied into cancer genomic subtyping or classification [31]. Due to the classification feature of SVM based on the large amounts of genomic data, SVM has been used to distinguish disease subtype [32], as well as discovery novel biomarkers or drug targets [31]. Similarly, this study also constructed a recurrent COAD discriminating classifier using SMV, which presented a good discriminatory capability for recurrent and non-recurrent COAD. Furthermore, the RS prognostic prediction model for recurrent COAD was constructed based on univariate and multivariate Cox regression analysis. Computationally, univariate and multivariate Cox regression were the most common method to construct the prognostic models and screen prognostic factors [33].

Co-expression network revealed that LINC01560 was co-expressed with *CXCL14* and *EPDR1*, and FOXP1-IT1 was co-expressed with *HEPACAM* and *ST6GALNAC1*. Moreover, FOXP1-IT1 was a protective factor while LINC01560 was a risky factor for COAD recurrence. Higher expression of LINC01560 was related to poorer survival, whereas upregulation of FOXP1-IT1 exhibited good survival. LINC01560 was reported to be aberrantly expressed in osteosarcoma [34]. *CXCL14*, termed breast and kidney expressed chemokine (BRAK), is one conserved chemokine involved in the activation of various immune cells such as natural killer cells, immature dendritic cells, and macrophages [35]. Consistent with our study, the report of Zeng et al. has found the significantly elevated expression level of *CXCL14* in colorectal carcinoma tissue compared with normal tissues, and high *CXCL14* expression increased the recurrence risk of colorectal carcinoma [36]. *EPDR1*, which encodes for type II transmembrane protein, is originally identified in teleost fishes and involved in cell adhesion [37]. Gimeno et al. have revealed upregulated *EPDR1* in colorectal carcinoma tissue, and *EPDR1* may be a potential biomarker of tumor invasiveness in patients with colorectal carcinoma [38]. However, whether lncRNA-*CXCL14*/*EPDR1* axis was associated with the survival of patients with COAD recurrence still needs to be investigated in the following analysis.

*HEPACAM* is originally discovered in the liver and then considered as one member of the immunoglobulin superfamily [39]. Recent studies have demonstrated *HEPACAM* was under-expressed in several cancers. A recent study has shown that *HEPACAM* was expressed at low level, and *HEPACAM* overexpression inhibited cell proliferation, migration, and invasion in colorectal cancer [40]. *ST6GALNAC1* is a key sialyltransferase for the biosynthesis of the cancer-associated Sialyl-Tn (STn) antigen that involved in cell adhesion, invasion, and metastasis in cancers [41]. Several studies have suggested that ST6GalNAc1 is overexpressed in breast and colon cancer, and upregulation of ST6GalNAc1 promotes tumor growth and metastasis [42, 43]. Unfortunately, the relationships between FOXP1-IT1-*HEPACAM*/*ST6GALNAC1* and prognosis of patients with COAD recurrence have not been elucidated.

We found that ZNF667-AS1 was another risky factor for survival evaluation of patients with COAD. Previous studies have demonstrated that ZNF667-AS1 was dysregulated in multiple cancers and associated with tumor invasion and metastasis [44–46]. Peng et al. recurrently constructed a lncRNA-related competing endogenous RNA network and highlighted that ZNF667-AS1 was a predictor for survival prognosis of gastric cancer [47]. Several researchers also suggested that LINC00174 played significant roles in the molecular pathogenesis of several cancers, including hepatocellular carcinoma and colorectal carcinoma [48, 49]. Our analysis revealed that the overexpression of ZNF667-AS1 was related to the poor prognosis of patients with COAD recurrence. However, the influences of ZNF667-AS1, LINC00174, LINC00852, and TAF1A-AS1 on COAD recurrence have not been completely illuminated. Therefore, we will collect more clinical information to explore the effects of combination of these lncRNA signatures on COAD. Moreover, corresponding function mechanisms were also required to investigate by in vitro and in vivo assays LINC00852, LINC01560, and LINC00174.

## Conclusion

In conclusion, this study constructed a SVM classifier for identifying recurrent COAD patients and prognostic prediction model for COAD recurrence. To our knowledge, this is the first study to screen six lncRNA signatures for predicting COAD recurrence, which is important to develop a novel therapeutic strategy for improving the survival of patients with COAD recurrence. However, the underlying mechanism of these lncRNA signatures should be further investigated.

## Data Availability

The data and materials are available under the permission of author.

## Abbreviations

COAD: Colon adenocarcinoma
lncRNAs: Long non-coding RNAs
NCBI: National Center for Biotechnology Information
TCGA: The Cancer Genome Atlas
RFE: Recursive feature elimination
SVM: Algorithm based on the support vector machine
AUC: Area under the curve
ROC: Receiver operating characteristic
Sen: Sensitivity
Spe: Specificity
PPV: Positive prediction value
NPV: Negative prediction value
RS: Risk score
K-M: Kaplan-Meier
DERs: Differentially expressed RNAs
DAVID: Database for Annotation, Visualization, and Integrated Discovery
GO: Gene Ontology
KEGG: Kyoto Encyclopedia of Genes and Genomes
